# Further Enhancement of Mechanical Properties of Conducting Rubber Composites Based on Multiwalled Carbon Nanotubes and Nitrile Rubber by Solvent Treatment

**DOI:** 10.3390/ma11101806

**Published:** 2018-09-23

**Authors:** Pasi Keinänen, Amit Das, Jyrki Vuorinen

**Affiliations:** 1 Tampere University of Technology, Laboratory of Materials Science P.O. Box 527, FI-33101 Tampere, Finland; das@ipfdd.de (A.D.); jyrki.vuorinen@tut.fi (J.V.); 2 Leibniz Institute of Polymer Research Dresden, P.O. Box 120 411, D-01005 Dresden, Germany

**Keywords:** CNT, NBR, nanocomposite, dispersion, post-treatment

## Abstract

Post-treatment removal of dispersion agents from carbon nanotube/rubber composites can greatly enhance the mechanical properties by increasing the filler–matrix interaction. In this study, multiwall carbon nanotubes (MWNT) were dispersed in water by sonication and nonionic surfactant, octyl-phenol-ethoxylate was used as a dispersion agent. The dispersed MWNTs were incorporated in thermo-reactive acrylonitrile butadiene rubber (NBR) latex and nanocomposite films were prepared by solution casting. As a post-treatment, the surfactant was removed with acetone and films were dried in air. Dispersion quality of the colloid before casting was determined, and mechanical, electrical and thermal properties of the composites before and after the acetone post-treatment were studied. It was found that removal of dispersion agent increased the storage modulus of films between 160–300% in all samples. Relative enhancement was greater in samples with better dispersion quality, whereas thermal conductivity changed more in samples with smaller dispersion quality values. Electrical properties were not notably affected.

## 1. Introduction

Elastomers are being reinforced with several different types of fillers to improve their mechanical, electrical and thermal properties. Typical fillers include sub-micron carboneous particles or silica depending on the application. There are many factors influencing the enhancements in composite properties including size distribution of the particles, aspect ratio, dispersion quality and adhesion to the matrix.

In last two decades, there has been a great effort to introduce novel nanosized particles in to elastomer matrices. These include nano-silica [1], organo-clays [2] and carbon with different morphologies like carbon nanotubes, graphene, etc. In theoretical speculations, one of the most promising nanosized fillers are carbon nanotubes (CNT) [3] due to their geometrical structure and intrinsic mechanical and electrical properties. Despite the high expectations, there have been many challenges in realizing their potential as filler in soft rubber matrix including achieving proper dispersion, mixing technology, no standard specification about the quality and type of the tubes.

Compared to many other fillers, carbon nanotubes forming large agglomerates are more challenging to use. To optimize the dispersion process, enough energy density needs to be generated to overcome internal forces holding the aggregates together. Typical methods include shear-mixing [4,5], and sonication [5,6]. From these, the sonication is superior especially in low viscosity systems where conventional mixing methods cannot create required high strain rates. The dispersion process using sonication is based on inertial cavitation where imploding microscopic cavities are generating intensive streams of molecules, which again creates high strain rates and energy densities inside the liquid, enough to break nanotube agglomerates in to smaller aggregates and detach individual tubes. These cavities are also known to preferably exist on boarder lines of different materials, which make sonication a very effective and precise method for dispersing nanosize particles [7]. It is important to optimize the sonication process since prolonged sonication however can cause damage and alter the properties of the tubes [8,9]

After the nanotubes have been detached from aggregates, there is a high possibility of re-agglomeration. To stabilize the system different types of surfactants are being used including sodium dodecyl sulfate (SDS) [10], sodium dodecylbenzene sulfonate (SDBS) [11] and octyl phenol ethoxylate (TritonX-100) [10]. Molecules act as buffers between separated nanotubes preventing their re-agglomeration since van der Waals forces, responsible for the phenomena, is decreasing very rapidly as a function of distance. Surfactants are therefore essential in stabilizing and improving a dispersion quality of the colloids, a factor greatly affecting the properties of many applications. However, they can also inhibit the physical interactions between fillers and the matrix in processed nanocomposites.

Reinforcing the matrix is directly related to good adhesion and to the percolating contact area of the individual nanotubes and therefore wrapping the tubes with surfactants constrains the enhancements. Different procedures have been presented to remove the excess surfactant after nanocomposite structures were manufactured including: nitric acid [12], Fenton reaction [13], photocatalysis [13], organic solvents [14] and water rinsing [15]. Most of the studies were performed on transparent conducting thermoplastic films or on cellulose structures and very little information is found regarding elastomer composites.

In this paper, we demonstrate a simple method to enhance physical properties of NBR/CNT nanocomposite films by removing the surfactant from the matrix with acetone. It was found that removal of dispersion agent increased the storage modulus of films over 150% in all samples. Relative enhancement was greater in samples with better dispersion quality. In addition, thermal conductivity increased by over 100% with lower dispersion quality samples. Electrical properties were not notably affected.

## 2. Materials and Methods

### 2.1. Materials

Aqueous dispersion of thermoreactive butadiene acrylonitrile copolymer (Litex®N2890, Synthomer plc, London, UK) was used to cast a matrix (Table 1), multiwall carbon nanotube powder (NC7000^TM^, Nanocyl SA., Sambreville, Belgium) was used as a filler (Table 2) and octyl phenol ethoxylate (Triton X-100, Merck KGaA, Darmstadt, Germany) was used as a dispersion agent.

### 2.2. Dispersion of Carbon Nanotubes

Fifteen samples with 0.40 ± 0.02 g of NC7000^TM^ multiwall carbon nanotubes, 0.40 ± 0.02 g of Triton X-100 and 79.20 ± 0.01 of deionized water was weighed in 100 mL glass beakers and sonicated using different acoustic energies. A concentration of carbon nanotubes was selected to be low enough to avoid dramatic increase in viscosity as CNTs start to disperse. Increase in viscosity would otherwise increase the acoustic resistance which could diminish the dispersive power of the process. For sonication, a tip sonicator (QSonica Q700, Qsonica L.L.C, Newton, CT, USA) with 12.7 mm diameter titanium probe and vibration amplitude of 120 μm was used. To guarantee identical sample preparation, the tip was always placed in the same position inside the beaker (15 mm ± 2 mm from the bottom) and external cooling bath with c. 200 W cooling capacity was used to limit the temperature variations during the sonication. Acoustic energy was monitored by internal calorimeter of the QSonica Q700 device and varied by controlling the sonication time. An acoustic power reading given by the sonicator remained between 110–140 W in all sonications.

### 2.3. UV-VIS Measurements

An opacity at 500 nm, directly related to a concentration of carbon nanotubes in dispersed state, was used to measure the quality of the sonicated MWNT-dispersions in water [16]. A portion of each dispersion was collected, settled for five days and its supernatant diluted with deionized water with the ratio of 1:300 to get the solutions transparent. The absorbance of the diluted dispersions was measured with spectrophotometer (Shimadzu UV-1800, Shimadzu Corp., Kyoto, Japan) using quartz cuvettes.

### 2.4. Casting of NBR/CNT Nanocomposites

After the dispersion quality as a function of sonication energy for MWNT dispersion was determined, selected colloids were prepared for casting. Five samples of 61.5 ± 0.1 g of CNT-solution with different dispersion qualities were mixed with 6.0 ± 0.1 g of NBR copolymer latex and left to dry for 72 h in flat 120 × 120 mm size acrylic molds. After drying, the films were cross-linked by heat in an oven for 3 h 100 °C. A CNT concentration in all samples was the same, 12.5 ± 0.3 phr and average thickness of the films was 200 ±20 μm. The concentration was chosen to be higher than a percolation threshold of NC7000^TM^ carbon nanotubes [17]. To remove surfactants as a post-treatment from the selected samples, films were immersed in acetone for 60 min and dried in room temperature.

Another similar set of films were fabricated for stress–strain measurements by repeating the solution casting steps three times increasing the film thickness to 600 ± 50 μm.

### 2.5. Surface Resistance Measurements

Electrical surface resistance of the NBR/CNT composites was measured before and after the post-treatment with an electrometer and a resistivity chamber (Keithley 6517 electrometer and 8009 Resistivity Chamber, Tektronix, Inc., Beaverton, OR, USA), and by following an ASTM D257 standard. CNT/NBR nanocomposite films were placed between electrodes and in plane current was detected at 3.0 V. The current was let to relax for 60 s after which the reading was made.

### 2.6. Thermal Conductivity Measurements

Through plane thermal diffusivity of the films before and after the post-treatment was determined with a laser flash analysis (LFA) method (Netzsch Hyperflash LFA 467, Erich NETZSCH GmbH & Co. Holding KG, Selb, Germany) following ASTM E1461 standard. In addition, 10 mm × 10 mm piece of the sample film was placed in a sample holder, sprayed with graphite paint and a through plane heat signal was detected at room temperature. The specific heat of the samples for LFA was determined in room temperature with a differential scanning calorimeter (DSC) (DSC 204 F1 Phoenix, Erich NETZSCH GmbH & Co. Holding KG, Selb, Germany) using standard ISO 11357 Part 4. In addition, 10 ± 0.1 mg of the film material was weighed in a crucible and heated from −60 ${}^{\circ }$C to 60 ${}^{\circ }$C with 10 ${}^{\circ }$C/min heating rate and using N${}_{2}$ as purge gas. Reference material for specific heat capacity measurements was single crystal sapphire. Heat exchange and temperature was monitored from which specific heat was calculated. A volume density of the samples was calculated from the volume and weight of the samples.

### 2.7. Stress–Strain Measurements

Stress–strain studies were performed with tensile tester (Messphysik midi 10–20, Messphysik Materials Testing GmbH, Furstenfeld, Austria) and dumb-bell test pieces. Selected crosshead speed was 100 mm per minute.

### 2.8. Dynamical Mechanical Analysis

Dynamic mechanical analysis was carried out using rectangular specimen using a dynamic mechanical thermal analyzer (Eplexor 150N, Gabo Qualimeter Testanlagen GmbH, Ahleden, Germany) in the tension mode. The isochronal frequency employed was 10 Hz and the heating rate was 2 ${}^{\circ }$C/min with a dynamic load of 0.2% strain and static load at 0.5% strain. The amplitude sweep measurements were performed with another dynamic mechanical thermal analyzer (Eplexor-2000N, Gabo Qualimeter Testanlagen GmbH, Ahleden, Germany) in tension mode at room temperature, at a constant frequency of 10 Hz, 60% pre-strain and dynamic strain from 0.01–30%.

## 3. Results and Discussion

### 3.1. UV-VIS Measurements

**Figure 1:**
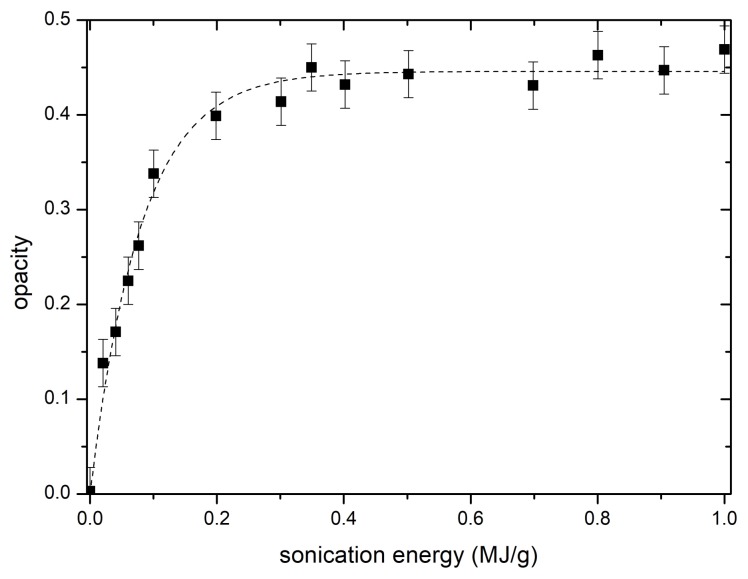
Opacity of 300 times diluted CNT colloid supernatant as a function of sonication energy per CNT mass with fitted logistic function.

Figure 1 shows that the opacity at 500 nm as a function of sonication energy follows a logistic curve (dashed line) deducted in the author’s earlier work [18]. Since opacity is related to dispersion quality, one can use it to optimize the sonication energy to prevent oversonication and damaging the tubes. For casting, five samples were selected representing different dispersion qualities and sonication energies from 0 to 0.4 MJ/g. In order to simplify future data analysis, a concept of relative dispersion quality (RDQ) was used referring to measured opacity of an individual sample divided by saturated opacity achievable by the system with infinite sonication energy.

### 3.2. Acetone Immersion

In order to remove free and bound surfactant from the films, they were immersed in acetone for 60 min. After drying, it was noticed that mass change was greater than the mass of added surfactant (Triton X-100). This was concluded to be caused by removal of surfactants of the pristine latex.

The masses of the films in Table 3 are not equal even the casting was done with equal masses. The reason is that, during the removal of films from the molds, part of the material was lost and only undamaged parts were processed further.

### 3.3. Surface Resistance Measurements

**Figure 2:**
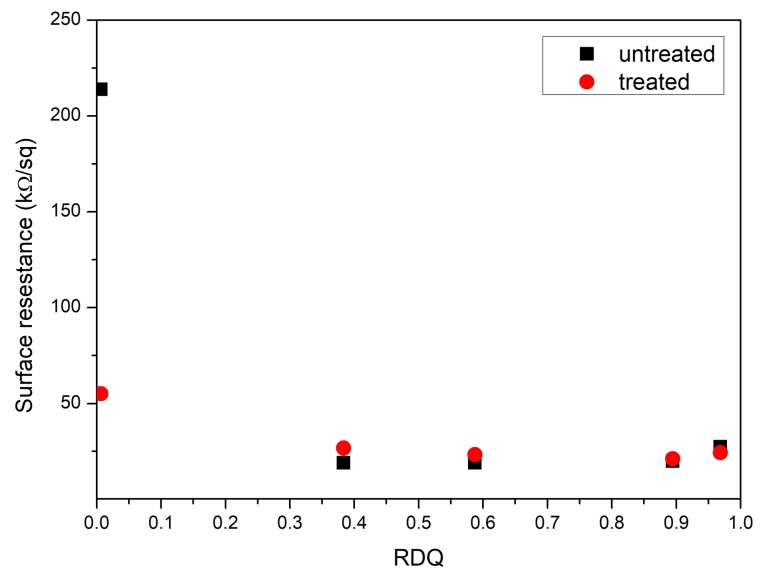
Surface resistance of untreated and post treated samples with different relative dispersion qualities.

After an initial drop, the surface resistance of the rubber sample did not notably change as a function of dispersion quality or post treatment (Figure 2). The resistance values are remaining in the range of 20 kΩ/sq to 30 kΩ/sq. The loading of the tubes in all samples is at 12.5 phr, so the concentration of carbon nanotubes is significantly above the critical percolating concentration. For this reason, the conductivity of the samples did not alter that much with different dispersion qualities after acetone treatment. Even at lower dispersion quality, the distribution of the CNTs took place in such a way that the nanotubes could form a percolating network. Surfactant absorbed on CNTs did not significantly increase the contact resistance of the percolating network.

### 3.4. Thermal Conductivity Measurements

Thermal diffusivity was determined as an average from 15 individual flashes. Thermal conductivity κ of the sample was calculated using relation, $\kappa =a\rho c_{p}$, where *a* is the thermal diffusivity, ρ is volume density and $c_{p}$ is the specific heat determined with DSC (0.75 ± 0.05 kJ/${}^{\circ }$C kg).

**Figure 3:**
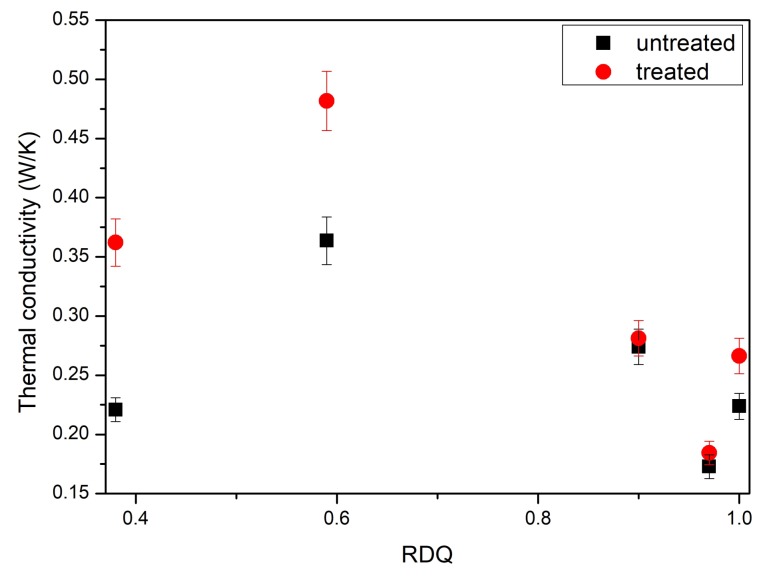
Thermal through-plane conductivity of untreated and post treated samples with different relative dispersion qualities.

Thermal conductivity peaked with relative dispersion quality close to 0.6 (Figure 3). In this value, the sonication process experienced some type of gelation point where loose CNT agglomerates start to percolate throughout the dispersion. At this point, the physical interaction of CNT network is at the highest level, which also leads to an increase in thermal conductivity. Acetone post-treatment enhanced the thermal conductivity with smaller dispersion quality values but was marginal with higher values.

### 3.5. Stress–Strain

**Figure 4:**
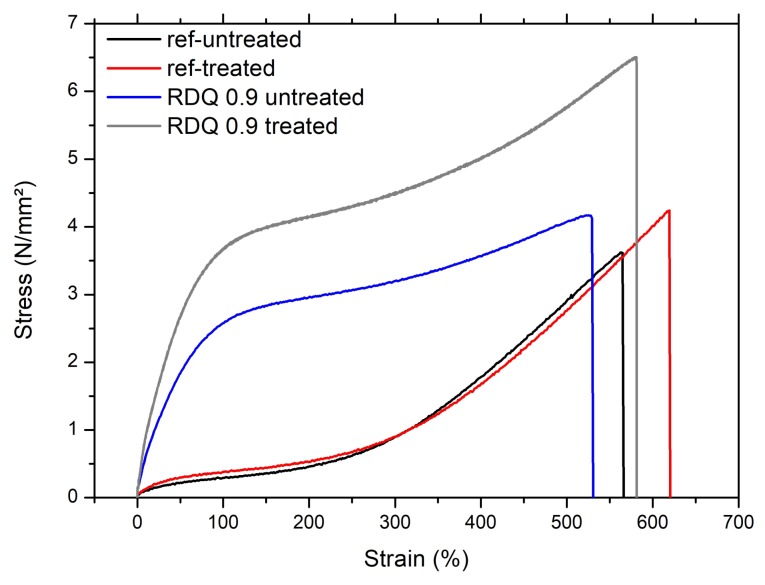
Comparison between untreated and treated nanocomposites with high relative dispersion quality vs. untreated and treated reference material.

The mechanical properties of the rubber composites were evaluated that were prepared with the pre-dispersed CNT solution. It can be found that the NBR behaves like a well crosslinked rubber matrix with elongation at break around 600%. This rubber sample (without any filler) was also treated with solvent and, after the treatment, the stress–strain properties were not altered that much. After incorporation of CNT, the mechanical properties were improved to a considerable extent. It can be found that the modulus of the composites was gradually increased with the increase of the sonication dosage i.e., better relative dispersion quality. To understand the effect more elaborately, the stress–strain diagram from one of the representative samples is shown in Figure 4. It is clear that a small amount of nanotubes reinforces the rubber matrix as the curves becoming steeper. Here, it is interesting to note that, after solvent treatment, the stress is more at a given strain. This could be explained by the way that the small molecules of surfactant (what was used to disperse the tubes in aqueous medium) are leached away during the solvent treatment process. Thus, this result indicates that a vulcanized rubber sample could be made more mechanically robust by suitable solvent treatment.

**Figure 5:**
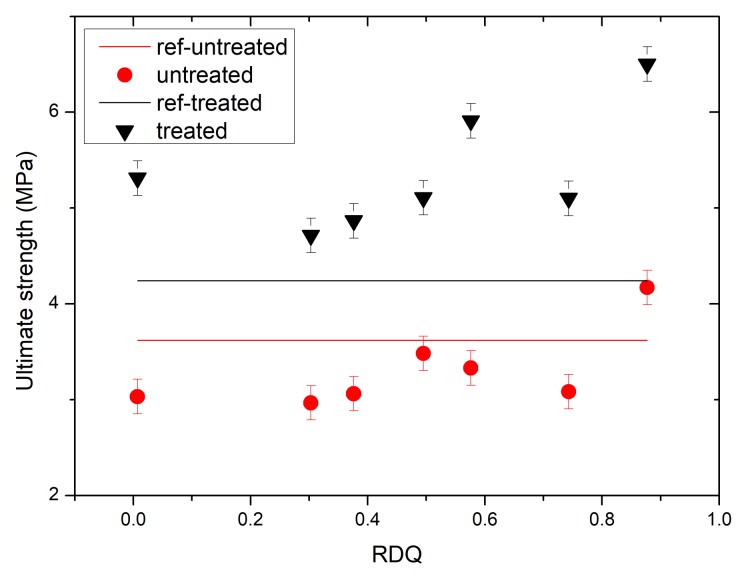
A development of ultimate strength of CNT/NBR nanocomposites as a function of relative dispersion quality. Dashed lines are representing unfilled NBR elastomer films.

From Figure 5, it can be seen that, as RDQ is increasing, the ultimate strength is also increasing. Before acetone treatment, the ultimate strength of the nanocomposite is below the untreated value of the pristine NBR film (lower dashed line), and after acetone treatment all ultimate strength values of nanocomposites are above the treated values of NBR (upper dashed line). One note again is to be made close to RDQ of 0.6 where the local peak is to be seen similar to thermal conductivity measurements.

Reference material is also being affected by the acetone treatment. This is due to the fact that the used Litex latex is also containing some surfactants that presumably are dissolved into acetone, improving the mechanical interaction of the rubber molecules.

**Figure 6:**
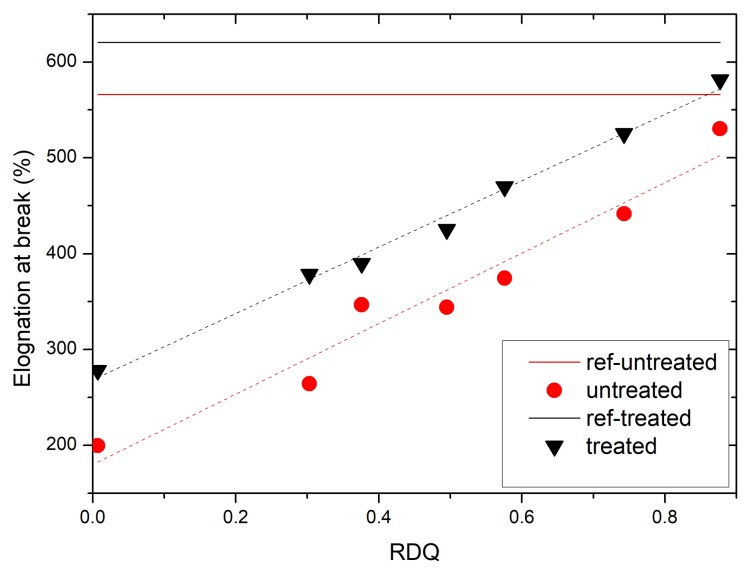
A development of elongation at break of CNT/NBR nanocomposites as a function of relative dispersion quality together with fitted dashed lines. Elongation at break is growing at slope 367% × RDQ in untreated composites and 346% × RDQ in treated composites. Solid lines are representing unfilled NBR elastomer films.

Figure 6 shows the development of elongation at the break of the films. As expected, the filled elastomers are more brittle compared to pristine rubber. However, in filled and treated elastomers, elongation at break is growing linearly as a function of RDQ and comes very close to unfilled NBR and over the untreated values of pristine film. It is therefore possible to manufacture stiffer elastomer composite that have the same properties of maximum elongation.

### 3.6. Dynamical Mechanical Analysis

The rubber composites were further investigated by strain sweep analysis to understand filler–filler interaction, which is generally called Payne effect. For filler containing cross linked rubber, the dynamic mechanic properties is largely dependent on the dynamic strain and this property is well known as the Payne effect. At higher dynamic strain, the filler–filler networks, developed inside the rubber matrix, is broken and a gradual fall of the storage modulus is noticed. However, a gum rubber without any filler does not respond with strain.

**Figure 7:**
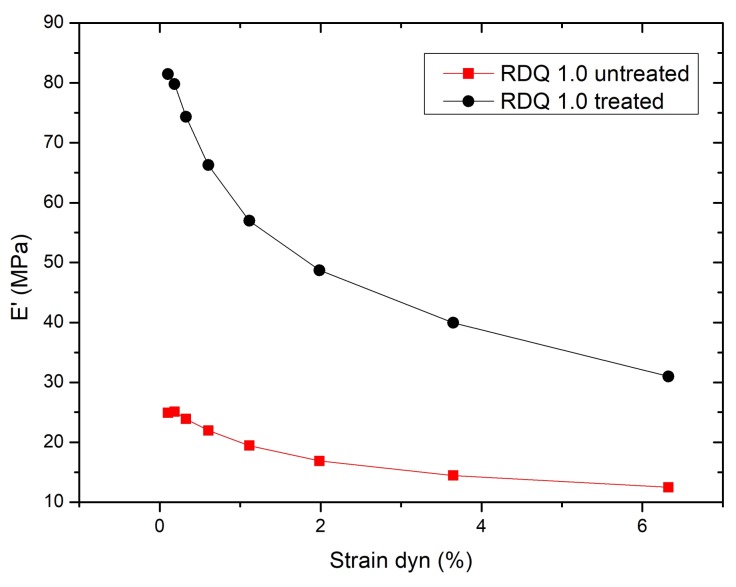
Payne effect of a CNT/NBR nanocomposites before and after the post-treatment.

**Figure 8:**
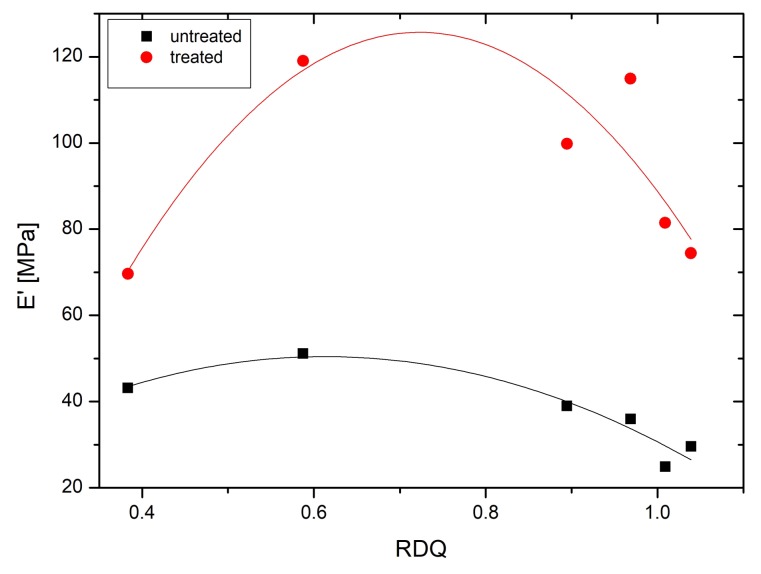
A development of storage modulus of CNT/NBR nanocomposites as a function of relative dispersion quality for untreated and treated films.

**Figure 9:**
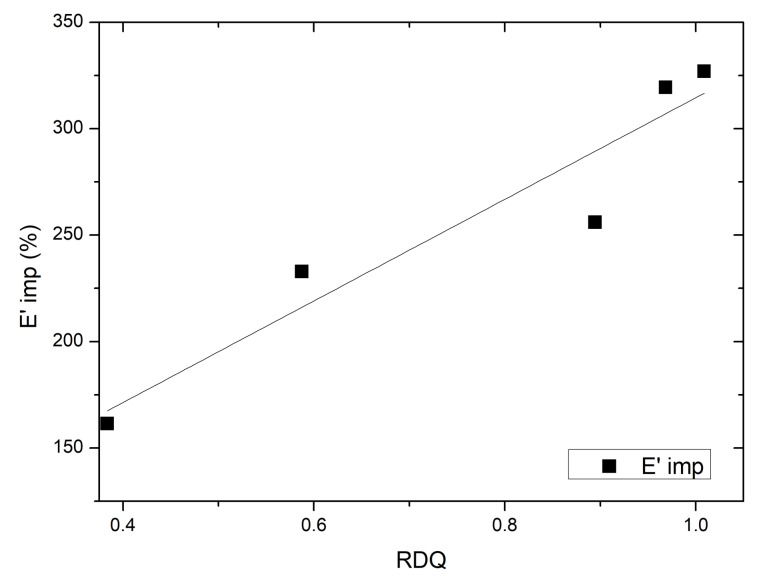
A development of relative improvement due to the post treatment of the storage modulus of CNT/NBR nanocomposites as a function of relative dispersion quality.

In the present case, the storage modulus ($E^{\prime }$) of the sample is plotted against dynamic strain (Figure 7). It can be seen that acetone post treatment enhanced the filler–filler interaction more than 300% in samples with higher RDQ. However, it can be seen that, with the increase of the filler dispersion, the dependencies of the storage modulus as a function of dynamic strain is not increasing linearly, but have again two local maximums at 0.6 with untreated and 0.7 RDQ with treated samples. (Figure 8). With higher sonication energy, the tubes are dissociated into finer particles and the native interaction of the CNT aggregates is lost. With RDQ close to 0.6, there seems to be an optimum where the pristine interaction from original aggregates is still present, and the aspect ratio of the particle is improved by the dispersion process. These two factors both have reinforcing effects on the composite. The relative improvement as a function of RDQ can be seen in Figure 9. It also very interesting to observe that, after solvent treatment, the dependency is more prominent, indicating more filler–filler interaction within the dispersed tubes. It can be envisaged that the surfactant molecules were initially deposited in between the tubes and were acting as plasticizers. After removing the surfactant, the two adjacent tubes are again coming closer and strong filler–filler interactions are realized. This trend also was observed with all other samples that are not shown here.

**Figure 10:**
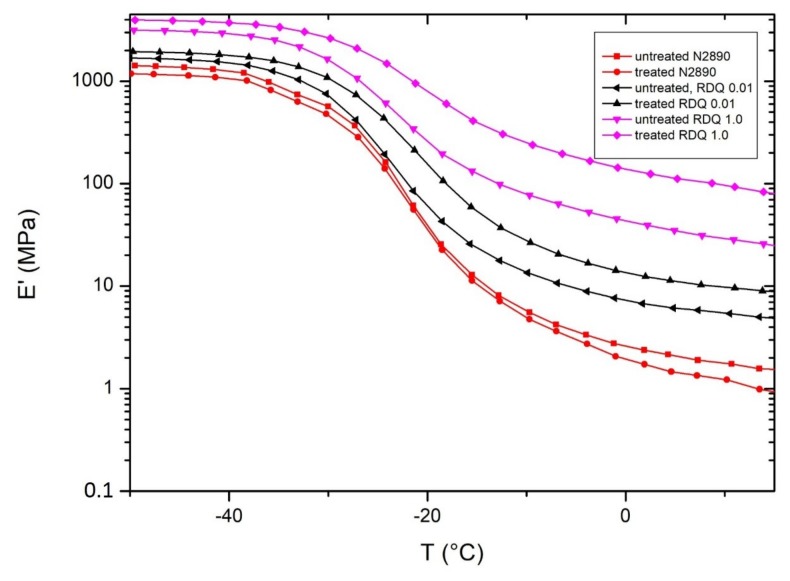
A development of storage modulus of CNT/NBR nanocomposites as a function of relative dispersion quality for untreated and treated films.

Dynamic mechanical properties of the samples were analyzed, a plot of storage modulus as a function of temperature was analyzed and the storage modulus values are tabulated, which were obtained at room temperature (rubbery plateau region). It can be seen that the storage modulus (above glass transition temperature) is increasing with the increase of dispersion quality of the CNTs (Figure 10. It should be noted here that the amount of the tubes is constant for all composites. In this case, higher storage modulus is associated with the reinforcing of the tubes to the soft elastomers. When the agglomerated structure of the tubes is dissociated under sonication conditions into fine individual tubes, the effective aspect ratio of the tubes is greatly enhanced and directly reflected in the reinforcing effect.

**Figure 11:**
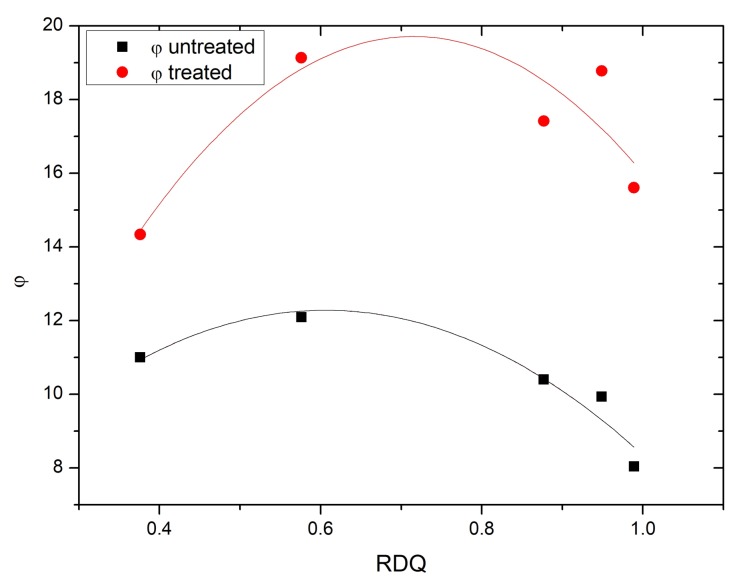
Shape factors, f, calculated using Equation (1), as a function of RDQ.

The Guth and Smallwood (GS) model was used to understand the reinforcing activity of CNT. Here, the shape factor f can be considered as as the ratio of length to thickness of the tubes in order to explain stiffening caused by chain-like structure or non-spherical particles (Equation (1)):

(1) $$\frac{E_{c}}{E_{m}}=1+0.67f\varphi +1.62f^{2}\varphi^{2},$$

where $E_{c}$ is Young’s modulus of the composites, $E_{m}$ is the Young’s modulus of the gum sample (without any filler) and φ is the volume fraction of the filler in the elastomeric composites. In the present case, the Young’s modulus is replaced by the storage modulus at a very low strain, $E^{\prime }\left(0\right)$, obtained from strain sweep analysis (Figure 7). In Equation (1), the φ is not constant but is dependent on size and shape of the particulate aggregates as stated by Sambrook [19]. A tightly bound interphasial volume between filler and the matrix is behaving more like a filler affecting the effective volume fraction of the filler-like domains. Using this approach and assuming no major decrease of the aspect ratio of the CNTs, the development of the effective volume fraction φ is shown in (Figure 11).

Calculating with Equation (1), the effective volume fractions are shown in (Figure 11). After an initial increase, φ starts to decrease as RDQ is increasing. It is interesting that again there is a peak close to RDQ value of 0.6 indicating strong total interaction. Acetone treatment is clearly enhancing the φ values of the model. An explanation to this could be that, as the interaction between filler and matrix is increased by removing the surfactant, an interphasial layer outside of the filler–matrix interface is gaining more filler-like characteristics, therefore changing the effective volume fraction and overall stiffness of the composite. This interphase is also dependent on the morphology of the filler particles. When filler morphology starts to get a more cylindrical shape by better dispersion quality, the overall interphasial volume is decreasing, since geometrical hindrance of the bound rubber is lost. This leads to decrease in the effective filler volume fraction and total reinforcement as can be seen in Figure 8.

## 4. Conclusions

Surfactants are perfect tools in non-covalent exohedral functionalization of carbon nanotubes. They leave the nanotubes undamaged during the mixing process and enable stable and high quality dispersion to be made. However, surfactants inhibit the realization of physical properties in nanocomposites by disturbing the interaction in filler networks and between fillers and matrix.

In this study, we have shown that simple acetone immersion after vulcanization removes residual surfactants from NBR/CNT nanocomposite films and improves their physical properties. Room temperature pre-strain storage modulus of the films increased more than 300% in some samples and storage modulus below glass transition, $T_{g}$ increased over 30%. We also saw that post treatment is affected more in samples with better dispersion quality. This is expected since better dispersion quality is related to increased concentration of adsorbed surfactant on a CNT surface. Removing surfactant enables better physical interaction between individual nanotubes and between tubes and the matrix. In our samples, the relative enhancements in mechanical performance of the nanocomposite films were significantly higher with post treatment than by using the CNTs as filler in the first place.

## Figures and Tables

**Table 1:** Physical properties of Litex®N2890 LATEX.

Property	Value	Unit	Method
Solids content	41.0	%	ISO 124
pH	7.3	-	ISO 976
Viscosity	<100	mPas	ISO 1652
Surface tension	31.9	mNm${}^{-1}$	DIN 1409

**Table 2:** Physical properties of NC7000^TM^ carbon nanotubes.

Property	Value	Unit	Method
Average diameter	10	nm	TEM
Average length	1.5	μm	TEM
Carbon purity	90	%	TGA
Transitional metal oxide	<1	%	ICP-MS
Surface area	250–300	m${}^{2}$g${}^{-1}$	BET

**Table 3:** Mass changes of films after acetone treatment.

RDQ	Mass of the Film (g)	Mass Difference From Acetone Treatment (g)
0.0	1.36	0.48
0.4	2.06	0.60
0.6	1.82	0.51
0.9	1.70	0.51
1.0	1.89	0.53
